# The Functions and Mechanisms of the Cohesin Complex in Regulating the Fate Determinations of Stem Cells

**DOI:** 10.34133/research.0757

**Published:** 2025-07-10

**Authors:** Jianghong Xiang, Yihan Lai, Zuping He

**Affiliations:** ^1^ Key Laboratory of Model Animals and Stem Cell Biology in Hunan Province, Engineering Research Center of Reproduction and Translational Medicine of Hunan Province, Institute of Interdisciplinary Studies, Hunan Normal University School of Basic Medicine, Hunan 410013, China.; ^2^ Key Laboratory of Reproductive Health Diseases Research and Translation, Ministry of Education of China, Hainan Academy of Medical Sciences, Hainan Medical University, Hainan 571199, China.

## Abstract

Stem cells have important applications in both regenerative and reproductive medicine. The cohesin complex comprises 4 core subunits, namely, SMC1, SMC3, RAD21, and STAG, and notably, it plays pivotal roles in controlling the fate determinations of stem cells by facilitating the dynamic regulation of the 3-dimensional genome architecture. We have recently reported that RAD21 forms a complex with YAP1 and NEDD4 to promote the self-renewal of human spermatogonial stem cells and inhibit their apoptosis. In this review, we address the molecular properties of the cohesin complex and its multiple regulatory mechanisms in mediating the fate decisions of various kinds of stem cells, including hematopoietic stem cells, embryonic stem cells, spermatogonial stem cells, neural stem cells, and other types of stem cells. By maintaining the chromatin loop structure, the cohesin complex is involved in DNA repair and gene transcription, which in turn controls the pluripotency, self-renewal, and differentiation of stem cells. In addition, the cohesin complex ensures faithful DNA replication and sister chromatid cohesion, which indirectly supports genetic and epigenetic programs. Variants in the subunit components of the cohesin complex and proteins’ modifications further confer functional plasticity, and its mutations can lead to abnormal stem cell functions and are correlated with diseases including cancers. Future studies need to integrate multidisciplinary approaches including single-cell multi-omics and cryo-electronic microscopy to resolve the dynamic regulatory networks of the cohesin complex in stem cell fate regulation and further explore its potential applications in regenerative and reproductive medicine.

## Introduction

As the core units driving tissue regeneration and homeostasis maintenance, stem cells and their fate-determination mechanisms have been a central focus in developmental biology and regenerative medicine. Traditional studies are involved in the regulation of stem cell fate decisions by transcription factors and signaling pathways [1,2]. However, a recent breakthrough in 3-dimensional (3D) genomics and epigenetics has revealed that dynamic chromatin topological reorganization plays an indispensable role in stem cell fate determinations. In this context, the cohesin complex, an evolutionarily conserved chromatin organizer, has emerged as a key regulator in stem cell biology, owing to its dual roles in mediating mechanical chromatin loop extrusion and indirectly shaping the epigenetic landscape.

The cohesin complex comprises SMC1/SMC3 heterodimer, RAD21, and STAG subunits, and it has initially been recognized for its classical function in sister chromatid cohesion [3–5]. Recent advancements in 3D genomics and functional studies have uncovered the noncanonical roles of the cohesin complex in different types of stem cells, including maintaining quiescence in hematopoietic stem cells (HSCs) [6,7], sustaining pluripotency in embryonic stem cells (ESCs) [8–11], and controlling fate decisions in neural stem cells (NSCs) [12–15]. The cohesin complex affects enhancer–promoter (E-P) interaction by mediating chromatin loop formation through its loop extrusion activity. These topological changes indirectly influence epigenetic states, e.g., SMC3 acetylation [16,17]. Notably, the cohesin complex exhibits context-dependent subunit composition (e.g., STAG1/SA1 vs. STAG2/SA2) and dynamically interacts with regulatory factors, including sororin (promoting cohesin) and WAPL (driving complex release) [18–22]. These interactions enable fine-tuned control of the chromatin architecture, which supports the lineage-specific functions of stem cells.

Notably, recent studies have shown that the aberrant functions of the cohesin complex can lead to an imbalance in stem cell homeostasis and that its dysregulation is associated with a variety of pathological processes, including myelodysplastic syndrome (MDS), acute myeloid leukemia (AML), and a number of other diseases. Mutations or aberrant posttranslational modifications of cohesin complex specific subunits can disrupt stem cell differentiation, trigger proliferative inabilities, and affect the maintenance of pluripotency. Therefore, regulating the assembly of cohesin complexes or remodeling chromatin topology may be a novel strategy to intervene in stem cell dysfunction.

Although canonical roles of the cohesin complex in ensuring chromosome segregation are well established, its functional landscape in stem cells remains incompletely mapped due to the following reasons. The context-dependent engagement of regulatory subunits (e.g., STAG1 vs. STAG2) and dynamic cohesin regulators (e.g., WAPL and sororin) in distinct stem cell types, e.g., spermatogonial stem cells (SSCs) vs. NSCs, has not been systematically addressed, especially regarding their activity modulation (e.g., posttranslational modifications) and functional divergence. The mechanistic links between cohesin-complex-driven chromatin topology and stem cell fate transition remain poorly understood. In this review, we discuss the recent advancements in the cohesin complex’s regulatory roles in stem cell biology, and notably, we propose a model in which the cohesin complex integrates microenvironmental signals to balance stem cell self-renewal and differentiation through dynamic chromatin restructuring and epigenetic modulation. By coordinating spatial genome organization with lineage-specific transcriptional programs, the cohesin complex establishes precise gene expression patterns critical for stem cell fate decisions. Therapeutic targeting of cohesin complex dynamics (e.g., by modulating loading factors) or subunit-specific interaction might provide novel strategies to manipulate stem cell plasticity, with great applications for regenerative medicine and cancer treatment.

## Structure and Functions of the Cohesin Complex

**Fig. 1.**
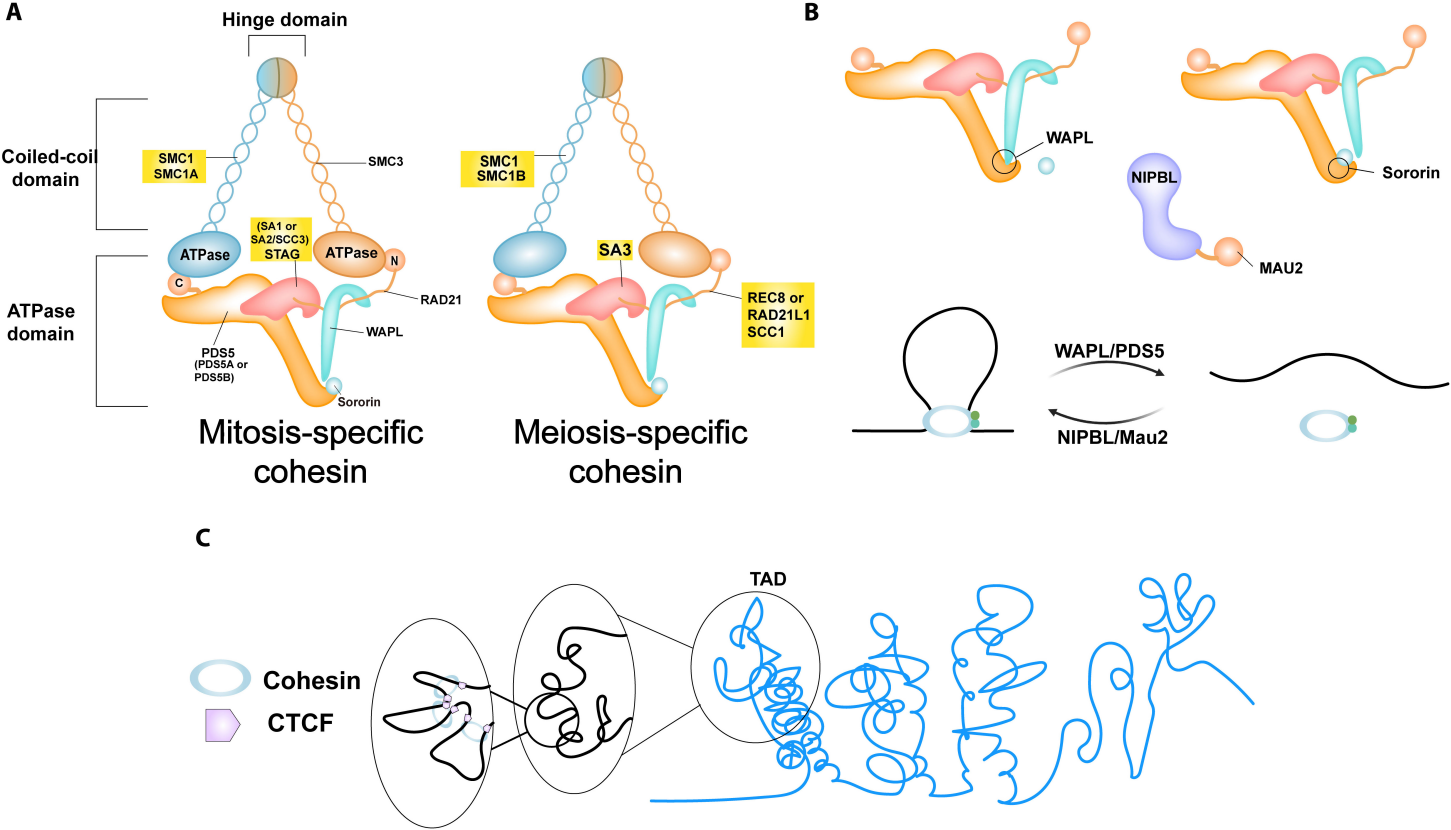
Structures of the cohesin complex. (A) Core structures of the cohesin complex. The complex cohesin is composed of 4 core subunits, namely, SMC1, SMC3, RAD21, and STAG, and there are mitosis-specific and meiosis-specific cohesin complexes. (B) WAPL negatively regulates sister chromatid cohesion by promoting cohesin dissociation from chromatin, whereas sororin stabilizes cohesin–chromatin binding by antagonizing WAPL function. The dynamic antagonism between these 2 modulators accurately regulates the loading/unloading balance of cohesin. (C) The cohesin complex synergizes with CTCF proteins to form chromatin ring structures that define topologically associating domain (TAD) boundaries to regulate 3-dimensional (3D) genome spatial organization.

The cohesin complex is evolutionarily conserved with 4 components, and SMC1 and SMC3 form a heterodimer through their hinge domains to create a flexible ringlike structure. RAD21 acts as a kleisin subunit bridging the SMC duplexes’ ATPase head to form a closed-loop topology, which serves as a platform for regulatory factors (e.g., WAPL) to modulate cohesin dynamics. SA subunits (STAG1/STAG2) work via dynamic regulatory modules, and their isoforms are selectively involved in different biological processes. For example, SA1 dominates 3D genomic reconfiguration in embryonic development [8,23,24], while SA2 is more inclined to maintain replication fork stability [24,25]. In contrast to SMC3, the remaining 3 subunits exist in multiple versions, and notably, SMC1β and STAG3 form functionally specific complexes adapted to germ-cell-specific requirement by replacing somatic subunits [26,27], as we illustrate in Fig. 1. The molecular architectures and regulatory mechanisms of the cohesin complex are shown in Table 1.

**Table 1.** Molecular architecture and regulatory mechanisms of cohesin complexes

Components	Molecules	Structural features	Functions	References
Core subunits	SMC1A (SMC1B)	N-terminal coiled coil, hinge domain, ATPase head domain with conserved motifs for ATP binding and hydrolysis	ATP-hydrolysis-driven DNA loop extrusion	[3]
	SMC3		Forms ATPase dimer with SMC1A; acetylation regulates dissociation	[37–39]
	RAD21 (REC8)	Acts as a kleisin subunit, bridging SMC1–SMC3 ATPase heads to form a closed-loop topology	Stabilizes SMC1A/SMC3 ring; serves as a cleavage target for separase during anaphase; stabilizes cohesin–DNA interactions	[44–46]
	STAG1/2(STAG3)	Exists as isoforms (STAG1/SA1 and STAG2/SA2); forms dynamic regulatory modules	Anchors cohesin to chromatin; mediates CTCF-dependent looping	[8,23–25]
Regulatory factors	NIPBL/MAU2	Forms a heterodimer (Scc2–Scc4 homolog in yeast); facilitates ATP hydrolysis	Loads cohesin onto DNA; promotes ring closure	[28–30]
	WAPL/PDS5	Forms a complex antagonized by sororin and PDS5; interacts with WAPL to promote cohesin release	Releases cohesin from DNA (ring opening); counteracts sororin to regulate dynamic equilibrium	[31–36]
Collaborating factors	CTCF		Anchors cohesin for loop formation and genome organization	[50–52]

The binding of the cohesin complex to chromatin is dependent on the ATP hydrolysis process, which is facilitated by the NIPBL–MAU2 heterodimer (Scc2–Scc4 in yeast) [28–30]. The double-stranded DNA is wrapped within the cohesin ring via a topological entrapment mechanism. This binding does not rely upon DNA-sequence-specific recognition, whereas it is achieved through a topological entrapping mechanism, thus conferring a broad genome-binding capacity. In terms of dynamic equilibrium, the WAPL–PDS5 complex forms a dynamic antagonistic system with sororin. WAPL–PDS5 promotes the cohesin unloading [31–33], whereas sororin antagonizes the unloading effect of WAPL, thereby stabilizing sister chromatid cohesion via competitively binding to PDS5 and recruiting PP2A phosphatase [34–36]. This regulatory network is further enhanced in the S phase by ESCO1/2-mediated modification of SMC3 acetylation, which ensures the structural integrity of chromosomes after replication [37–39].

The cohesin complex regulates chromatin architecture and maintains sister chromatid cohesion by topologically entrapping DNA within its ring structure. This process is vital for safeguarding mitotic and meiotic fidelity. During the S phase (or G1 in yeast), the NIPBL–MAU2 heterodimer relies on ATP hydrolysis to load the cohesin onto one single chromatid, which forms the initial binding framework. Following the completion of DNA replication, the complex wraps around the sister chromatids through a ring structure, and its stability is facilitated by the acetylation of the SMC3 subunit (catalyzed by ESCO1/ESCO2) and the binding of sororin to PDS5, which antagonizes the WAPL–PDS5-mediated dissociation of the complex. This mechanism is of particular significance in complex regions, e.g., telomeres, since it prevents genomic break caused by replication fork collapse [24,25].

**Fig. 2.**
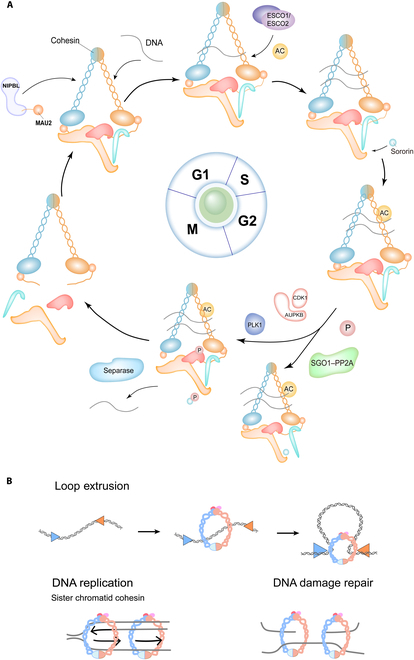
Dynamic regulation of the cohesin complex in the cell cycle of stem cells. (A) The cohesin complex is involved in regulating the G1, S (DNA synthesis), G2, and M (mitosis) phases of stem cell cycles by the genes *ESCO1*/*ESCO2*, *Sororin*, *CDK1*/*PLK1*, and *Separase*. (B) The cohesin complex supports the 3D structure of the genome by mediating the extrusion of chromatin loops, adhering sister chromatids to ensure accurate segregation after DNA replication, and maintaining their physical linkage to support homologous recombination repair during DNA damage repair.

During mitotic entry, CDK1, AURKB, and PLK1 initiate the prophase by phosphorylating sororin and SA subunits, which leads to WAPL–PDS5-complex-mediated removal of cohesin from chromosome arms [40,41]. Concurrently, the complex at the centromere is protected by the SGO1–PP2A complex, which dephosphorylates cohesin subunits to counteract prophase kinase activity to preserve sister chromatid linkage until anaphase [42,43]. Eventually, the separase cleaves the RAD21 subunit to complete sister chromatid separation, a process strictly limited by the anaphase-promoting complex/cyclosome (APC/C)-regulated securin degradation cascade that prevents aneuploidy from arising [44–46], as we summarize in Fig. 2.

As a highly conserved and multifunctional complex in eukaryotes, the biological roles of the cohesin complex are far beyond sister chromatid cohesion. Its multifunctionalities arise from context-dependent partnership with regulatory proteins (e.g., CTCF and NIPBL [47,48]), which enable 3 core mechanisms. Firstly, the cohesin complex collaborates with CTCF to form chromatin loops and maintain topologically associating domains (TADs), primarily through its SA1 subunit [49–52]. Secondly, the cohesin complex facilitates dynamic mediation of E-P interaction to coordinate gene transcriptional programs [53–55]. Thirdly, the cohesin complex stabilizes replication forks by topologically linking sister chromatids, which prevents fork collapse and ensures error-free restart [56–59].

In stem cells, the functions of the cohesin complex are further enriched. The cohesin complex not only maintains the structural integrity of the genome but also directly regulates the balance between stemness maintenance and lineage differentiation by integrating epigenetic modifications and microenvironment or the niche signals. In the following sections, we systematically address the regulatory network of the cohesin complex in different types of stem cells.

## The Mechanisms of the Cohesin Complex in Regulating the Fate Determinations of Various Kinds of Stem Cells

The specific mechanisms of the cohesin complex vary in different stem cells, and we summarize its regulatory networks and disease-related information in Table 2.

**Table 2.** Context-dependent functions and related diseases of the cohesin complex in stem cells

Stem cell types	Core functions	Regulatory networks	Disease linkage
HSCs	Maintain chromatin accessibility and E-P loop dynamics	STAG2 mediates CTCF-independent E-P loops for cell differentiation genes (Ebf1/Pax5) [60]	MDS/AML progression: *STAG2* mutations destabilize E-P loops and promote GMP expansion [61–63]; RAD21 haploinsufficiency activates HOX pathways [6,64]
	Epigenetic silencing and gene repression	RAD21 anchors H3K27me3 via PRC2 to suppress self-renewal genes (*HOXA7*/*HOXA9*) [64]	Leukemogenesis: SMC3 dosage imbalance (haploinsufficiency enhances self-renewal; complete loss causes ROS-driven exhaustion) [66–70]
	Metabolic-quiescence balance	The SMC3–YY1 complex represses Slc2a3 to maintain HSC quiescence [7]	Inflammation-driven clonal expansion: The RAD21/NF-κB feedback loop supports *STAG2*-mutant clones [71,72]
	DNA repair and genomic stability	SMC3/RAD21 stabilizes sister chromatid cohesion and replication forks [74,75]	Therapeutic targets: PARP inhibitor sensitivity (HRR defects) [77]; HDAC8 inhibitors restore SMC3 acetylation [17,67]
ESCs	Chromatin loop and TAD dynamics	SMC1A hinge domain maintains short-range loops; SA1/SA2 regulates TAD boundaries and PRC1 domains [79–81]	AML association: *SMC1A*-*R586W* mutation disrupts Oct4/Nanog expression [79]
	Pluripotency–differentiation balance	RAD21 colocalizes with Oct4/Nanog at super-enhancers; STAG2 drives naive-primed transition via Lin28a [8,23]	Differentiation defects: RAD21 loss induces DNA hypermethylation (DNMT3b up-regulation) [10]
	Epigenetic plasticity regulation	Oplr16 recruits TET2 to Oct4 promoter for DNA demethylation [84]	Reprogramming defects: Cohesin depletion triggers DNA damage and impairs pluripotency [11]
	Genomic stability maintenance	REC8/STAG3 loss increases replication stress and RPA foci [27]	Oncogenic risk: RetSat deficiency disrupts cohesin loading and promotes ESC tumorigenicity [86]
NSCs	Proliferation–differentiation balance	PHF2–RAD21 maintains TADs to suppress dormant replication origins [14]	Neurodevelopmental defects: STAG2/SMC1A knockout up-regulates forebrain genes (*ZIC2*/*GLI2*) [12]
	Mitotic fidelity	HNRNPA3–SMC1A regulates chromosome segregation; STAG1/STAG2 govern distal/local loops [13,15]	Brain disorders: Mitotic delay impairs cortical development [13]; TYW5 dysregulation links to psychiatric diseases [91]
SSCs	Self-renewal maintenance	RAD21–YAP1 regulates NEDD4 transcription to enhance self-renewal of human SSCs and suppress their apoptosis [92]	Male infertility: YAP1/RAD21/NEDD4 axis dysfunction in non-obstructive azoospermia [92]
ISCs	Stemness maintenance	Deletion of RAD21 leads to aberrant activation of *Esg* target genes (differentiation-related genes) [94]	Premature differentiation: RAD21 loss activates TGFB1/ITGA5; APC loss exacerbates genomic instability [94,95]

HSCs, hematopoietic stem cells; E-P, enhancer–promoter; GMP, granulosa–monocyte progenitor; PRC2, polycomb repressor complex 2; ROS, reactive oxygen species; PARP, poly(ADP-ribose) polymerase; HRR, homologous recombination repair; ESCs, embryonic stem cells; PRC1, polycomb repressive complex 1; AML, acute myeloid leukemia; RPA, replication protein A; NSCs, neural stem cells; SSCs, spermatogonial stem cells; ISCs, intestinal stem cells; APC, anaphase-promoting complex

### The regulation of the cohesin complex in HSCs

**Fig. 3.**
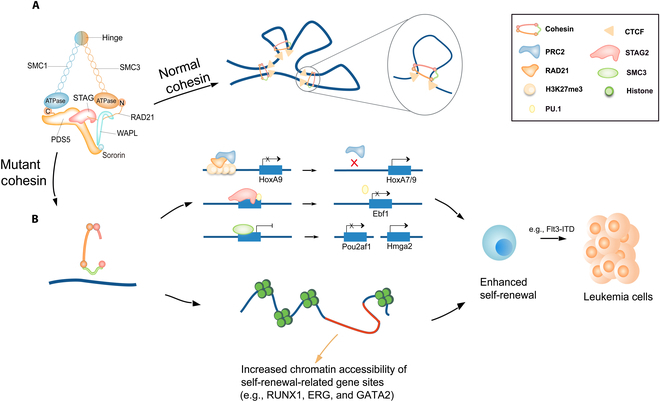
Functions and mechanisms of the cohesin complex regulation in HSCs and the development of AML. (A) Normal cohesin complex in controlling loop extrusion, DNA replication, and DNA repair of HSCs. The cohesin complex is involved in the construction of 3D genomic structures (e.g., TADs) and the regulation of gene expression by dynamically forming and promoting the extension of chromatin loops through loop extrusion. The cohesin complex also ensures DNA replication accuracy by stabilizing replication forks and promotes homologous recombination through the maintenance of sister chromatid cohesion in DNA damage repair, thereby safeguarding genome integrity in HSCs. (B) Mutant cohesin complex in mediating HSC fate decisions and diseases. The mutant cohesin complex affects the expression and chromatin accessibility of HSC self-renewal and differentiation genes, which leads to an enhancement of HSC self-renewal. The mutations of the cohesin complex result in AML.

The cohesin complex, in conjunction with its regulatory subunits, establishes a multilevel regulatory system that controls the fate determinations of the HSCs. This system works by means of dynamic remodeling of the 3D structure of chromosomes and integration of epigenetic modifications and signaling pathway networks. The regulatory system plays essential roles in mediating the self-renewal, differentiation, and genomic stability of HSCs, and it is also closely related to stress response and malignant transformation as we indicate in Fig. 3.

#### Synergistic regulation of chromatin topology and epigenetics in HSCs

The cohesin complex, a dynamic regulator of the 3D architecture of chromatin, collaborates with CTCF proteins to construct TADs and mediate the formation of E-P loops. This is achieved through the mechanism of DNA loop extrusion, thereby enabling the precise shaping of the spatial organization of the genome. In HSCs, STAG2 specifically facilitates CTCF-independent E-P loop formation, and its dynamics directly regulate chromatin accessibility. For key B-cell differentiation genes, e.g., *Ebf1* and *Pax5*, STAG2 ensures the normal initiation of the lymphoid lineage differentiation of HSCs by maintaining the chromatin-open state of the *Ebf1* site and the binding capacity of the PU.1 transcription factor [60]. In the absence of STAG2, the E-P loops of these critical regions dissociate, which triggers chromatin space compression and forces HSCs into the state of self-renewal enhancement and differentiation blockage. This ultimately leads to a lineage bias toward myeloid lineage development. This lineage bias is particularly pronounced in MDS-STAG2-mutant clones, which drives the malignant expansion of granulosa–monocyte progenitors and the transformation of these cells to AML by destabilizing the E-P loop in the CTCF deletion region and down-regulating transcriptionally pausing genes (e.g., *RUNX1* targets) [61–63].

In steady-state conditions, the cohesin complex is responsible for regulating the accessibility and expression status of the HSC genome by maintaining the 3D structure of chromatin. RAD21, a core subunit of the cohesin complex, has been shown to play a pivotal role in anchoring the H3K27me3 modification to the promoter region of stemness genes, such as *HOXA7*/*HOXA9*, thereby repressing their expression. This anchoring is achieved through its physical interaction with polycomb repressive complex 2 (PRC2). In normal HSCs, this epigenetic silencing mechanism effectively prevents activation of self-renewal-related genes, thereby inhibiting overproliferation of HSCs [64]. However, in the context of RAD21 haploinsufficiency, the recruitment efficiency of PRC2 is reduced, which results in HOX pathway activation and an enhancement in the regenerative capacity of HSCs. This phenomenon is further exacerbated by dysregulation of inflammation-related signaling pathways, such as the interferon α/γ-responsive pathway and IL2–STAT5 signaling pathway, which leads to an increased propensity for myeloid differentiation [6]. The imbalance in the epigenetic regulatory network is also a novel mechanism for the occurrence of leukemia in AML patients with cohesin mutations [64]. Concurrently, the mutant cohesin complex compromises the differentiation of HSCs by regulating chromatin accessibility and the activity of transcription factors (e.g., ERG, GATA2, and RUNX1), which may result in leukemia [65].

The dynamic equilibrium between metabolism and the resting state is predominantly associated with the cohesin complex subunit SMC3. SMC3 has been demonstrated to regulate the metabolic-resting equilibrium of HSCs through a dose-dependent mechanism. Its loss of function manifests the substantial nonlinear effect. Haploinsufficiency of *SMC3* activates the pluripotency network and inhibits differentiation [66,67], while complete deficiency of *SMC3* leads to the collapse of the HSCs’ function [67,68]. Under steady-state conditions, SMC3 forms a complex with the transcription factor YY1, thereby repressing its expression and maintaining the hypometabolic resting state of HSCs by inhibiting the expression of metabolic genes, such as *Slc2a3* [7]. In the context of haploinsufficiency, a decline in SMC3 expression has been observed to enhance the self-renewal of HSCs by reducing the chromatin accessibility of differentiation-related genes (e.g., *Pou2af1*), increasing the pluripotency network (e.g., Hmga2 and Pbx3), and synergistically promoting the genesis of AML in conjunction with Flt3-ITD. Furthermore, complete loss of SMC3 has been shown to trigger a burst of reactive oxygen species in mitochondria and defect in DNA repair, which ultimately leads to hematopoietic failure [66,69]. This dose-sensitive effect is particularly obvious in Down syndrome-associated AML-SMC3 deficiency that synergizes with *GATA1s* mutations to promote immature megakaryocyte expansion by activating the Toll-like receptor pathway [70].

#### Environmental interaction and DNA damage response of the cohesin complex in HSCs

The cohesin complex has been shown to form a dynamic network of interaction with inflammatory signaling pathways, and its function undergoes obvious reprogramming in response to inflammatory stimuli from homeostatic maintenance to stress-responsive regulation. In the context of acute inflammatory conditions, RAD21 has been shown to mediate the inflammatory response in senescent HSCs by enhancing the chromatin accessibility of NF-κB target genes (e.g., *IL6* and *TNFα*), which drives them toward the differentiated state to prevent the depletion of their resting pools [71]. Notably, RAD21 haploinsufficiency has been observed to reverse this phenotype and maintain the HSC resting state [6]. However, in chronic inflammation, the RAD21/NF-κB pathway is sustained, which results in a positive feedback loop. This loop involves the senescence-associated secretory phenotype, which in turn elevates the local microenvironmental levels of inflammatory factors. Consequently, this forces HSCs to enter a sustained cycle of proliferation and differentiation. This cycle is characterized by the formation of clones with cohesin mutations (such as *STAG2* deletion) that are selectively amplified. These cohesin mutations drive the transition of HSCs to a state of granulocyte–monocyte differentiation, and cells shift from dependence on the BCL2-mediated anti-apoptotic pathway to a survival pathway that relies on the TNFα-induced NF-κB signaling pathway to proliferate and escape apoptotic pressure [72].

In the DNA damage response, the cohesin complex precisely repairs double-strand breaks by facilitating homologous recombination repair (HRR) while stabilizing the stalled replication forks to restart DNA synthesis [64,73]. Its core subunit, SMC3, synergizes with RAD21 to mediate sister chromatid cohesion, which ensures the physical attachment of chromosomes after DNA replication and prevents genomic breaks caused by replication fork collapse [74,75].

#### Therapeutic strategies by using the cohesin complex in hematopoietic cancer

Mutations in the core subunits and regulatory factors of the cohesin complex (e.g., WAPL and PDS5) have been widely found in MDS and AML. This phenomenon reflects its central regulatory position in hematopoietic malignant transformations and provides a molecular basis for targeted therapies. Firstly, for the regulation of chromatin topology, it is expected to improve the clinical outcome of cohesin complex mutation-associated hematological malignancies by exploring the targeted chromatin ring dynamics (e.g., E-P ring stabilizers) or synthetic lethal strategies. It is noteworthy that STAG1 and STAG2 exhibit functional redundancy (e.g., maintenance of HSC survival [76]) and specificity (e.g., unique regulation of B-cell differentiation by STAG2 [60]) in HSCs. It may provide a rationale for the high prevalence of *STAG2* mutations in myeloid malignancies. The selective disruption of differentiation checkpoints while retaining the self-renewal capacity of HSCs by STAG2 provides a niche for malignant clonal evolution [62,75], which in turn offers a choice for targeted therapy. Based upon this biological property, the inhibition of STAG1 or blockade of WAPL-mediated cohesin complex unloading in STAG2-deficient AML cells leads to the breakdown of the chromatin structure and the selective removal of malignant clones. Furthermore, the synthetic lethal effect of DNA repair defect can also be exploited. For example, defective HRR due to cohesin deficiency increases sensitivity to poly(ADP-ribose) polymerase inhibitors, e.g., olaparib [77]. Further studies have demonstrated that combination with HDAC8 inhibitors (e.g., PCI-34051) reverses the aberrant SMC3 acetylation caused by ZNF521 overexpression and restores cell cycle regulation. Moreover, HSCs carrying *STAG2* and *SMC3* mutations are sensitive to treatment with azacitidine, a hypomethylating agent [17,67]. Therapies targeting the cohesin complex can be developed to be directed against its regulatory network rather than just individual subunit inhibition. For example, while selectively disrupting chromatin loops in leukemia cells using STAG2 inhibitors [62], strategies could be developed to dynamically control RAD21 cleavage by modulating separase activity to enhance HSC regeneration [78].

#### The challenges and future directions of the cohesin complex in HSCs and hematopoietic-related cancer

Although great progress has been made on the molecular mechanisms underlying the cohesin complex regulatory network, the spatiotemporal dynamics and interaction of the cohesin complex with the microenvironment remain largely to be further explored. Specifically, the dosage effect of SMC3 suggests that even minor expression reduction destabilizes cohesin’s chromatin loading and predisposes HSCs to stress-induced exhaustion [66,67], while the functional redundancy of STAG1/2 implicates that dual inactivation induces synthetic lethality in *STAG2*-mutant cancer models [76]. Furthermore, the synergistic remodeling of HSC fate by the inflammatory microenvironment and cohesin mutations (e.g., feedback regulation of the RAD21/NF-κB pathway by senescence-associated secretory phenotypes [71]) remains to be an important gap in understanding the transformation of clonal hematopoietic disease to leukemia. Notably, such an interaction may reshape HSC fate determinations through epigenetic reprogramming, but a systematic mechanistic resolution is lacking. To address these challenges, future studies can be directed toward integrating single-cell multi-omics and CRISPR/Cas13 screening [67,76] to systematically resolve the spatiotemporal-specific effect of the cohesin complex’s mutant subunits on specific subpopulations of HSCs. Furthermore, at the level of model construction, the combination of organoids, cryo-electronic microscopy, and in vivo imaging technologies has been expected to resolve the real-time assembly pattern of the cohesin complex. For the translational medicine of the cohesin complex, precise therapeutic strategies would be developed in terms of the dual dimensions of chromatin topology and epigenetics.

In summary, the cohesin complex plays pivotal roles in the fate determination of HSCs by means of the dynamic regulation of the chromatin structure, epigenetic status, and signaling pathways. Abnormalities in its function may result in genomic instability, differentiation defects, and hematopoietic malignant transformation, thus providing potential targets for precision therapy. On the other hand, the complexity of cohesin complex regulatory networks also implies that future studies are required to further resolve their spatiotemporal dynamics to develop more effective intervention strategies for these diseases.

### The regulation of the cohesin complex in ESCs

**Fig. 4.**
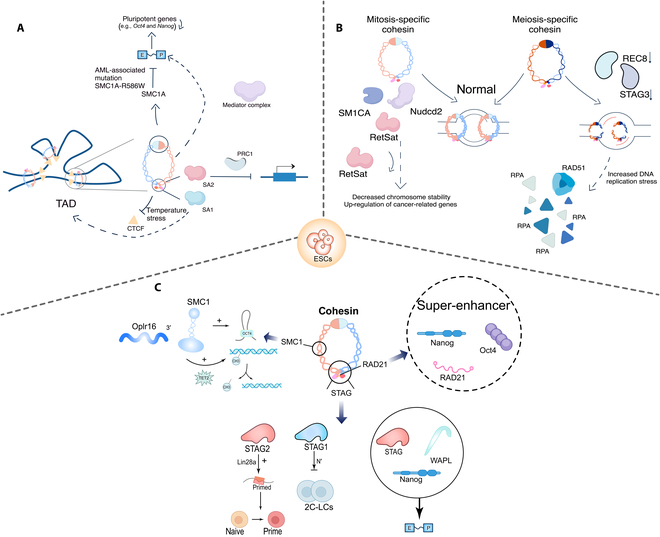
Functions and mechanisms of cohesin complex regulation in ESCs. (A) The cohesin complex dynamically regulates genome topology and chromatin loops. (B and C) The cohesin complex affects the genomic stability (B) and transcriptional regulation (C) of ESCs by regulating the genomic topology, E-P interaction, and epigenetic state. 2C-LCs, 2-cell-like cells.

The cohesin complex and its subunits construct a multilevel and regulatory system in the maintenance of pluripotency and fate decisions of ESCs by dynamically integrating chromatin 3D architecture, epigenetic modifications, and signaling pathway networks, as we illustrate in Fig. 4.

#### The dynamic regulation of genome topology and chromatin loops by the cohesin complex in ESCs

The spatial folding of chromatin is the physical basis for the spatiotemporal regulation of genes, and the cohesin complex plays a central role in mediating this process. Specifically, the cohesin shapes the 3D conformation of the genome through the formation of DNA loops and TADs, but its function extends far beyond structural maintenance. At the level of short-range chromatin interaction, the hinge domain of the SMC1A subunit has been shown to be a key regulatory element for short-range loop formation in ESCs. ESCs carrying the AML-associated mutation *SMC1A*-R586W exhibit diminished P-E interaction, which leads to dysregulation of the expression of pluripotent genes (e.g., *Oct4* and *Nanog*) [79]. This structural defect has been found to be correlated with the impaired CTCF-bound insulator function, reflecting that the hinge structural domain of the cohesin complex may influence the loop extrusion process by regulating the DNA-binding capacity of the complex [79]. At the level of long-range genomic organization, high-resolution mapping of chromatin interaction reveals that the cohesin synergistically anchors long-range constitutive interaction with CTCF to form the topological backbone of TADs, and the binding to the Mediator complex drives E-P junction within the submegabase scale and maintains transcriptional activity [80]. It is noteworthy that the regulation of the chromatin structure by cohesin SA1 and SA2 isoforms is scale dependent, with SA1 primarily maintaining TAD boundaries and SA2 repressing lineage-specific genes by promoting PRC1-mediated compression of multi-comb structural domains [81]. This “division of labor” pattern may explain why pluripotency genes are maintained but differentiation is severely impaired in SA2-deficient ESCs. Notably, the functional differentiation of this subunit is particularly pronounced in response to stress. For instance, temperature stress leads to changes in the binding sites of CTCF and cohesin, thereby affecting chromatin loop remodeling and regulating gene expression and the 3D chromatin structure [82]. Notably, the cohesin complex maintains the balance between self-renewal and differentiation of ESCs by regulating gene expression and influencing the chromatin structure, and the knockdown of its subunits RAD21 and REC8 induces the differentiation of ESCs into specific lineages, e.g., neural/germinative and endothelial cells, respectively [83].

#### Synergistic hub of transcriptional regulation by the cohesin complex in ESCs

The specific transcriptional program of ESCs not only is dependent on the binding of transcription factors but also requires the dynamic adaptation of the chromatin structure as physical support. It has been shown that the cohesin complex forms tight functional modules with pluripotent factors, and it plays a pivotal role in epigenetic–transcriptional coupling. First of all, the RAD21 subunit is involved in pluripotency through a nonclassical mechanism. It has been demonstrated that RAD21 is not dependent on CTCF and it colocates in the super-enhancer region with core transcription factors, e.g., Oct4 and Nanog, to directly maintain the active transcription state of pluripotency genes. When RAD21 is silenced, ESCs exhibit transcriptomic changes similar to *Nanog* deletion, reflecting a functional coupling between these 2 genes in the regulatory network. Further studies reveal that Nanog physically binds to STAG1 and WAPL, and this interplay may affect E-P junction by regulating the residence time of cohesin in chromatin [8]. The intricacy of this regulatory network is further supported by findings showing that the long-stranded noncoding RNA Oplr16 promotes the cyclization of chromatin at the Oct4 locus through the recruitment of SMC1 at its 3′ end while synergizing with TET2-mediated DNA demethylation to form a positive feedback loop of transcriptional activation [84]. This synergistic action of chromatin factors and epimerase modification highlights the bridging function of the cohesin complex in the epigenetic–transcriptional coupling of ESCs.

It is noteworthy that the heterogeneity of cohesin complex subunits has a substantial impact on their transcriptional regulatory specificity. While STAG1 and STAG2 are located in the same genomic region, they exhibit substantial functional differences. Specifically, STAG2, an important regulator of the transition between the naive and primed states of pluripotent stem cells, has been shown to enhance the expression of primed marker genes by activating *Lin28a* transcription and promoting its cytoplasmic localization. This process has been identified as a key driver of the transition of mouse ESCs from the naive state to the primed state. In contrast, the N-terminal end of STAG1 has been found to inhibit 2-cell-like cellular program by stabilizing the nucleolus structure [23,85]. Collectively, these findings suggest that the combinatorial diversity of cohesin complex subunits provides a molecular basis for their involvement in the fate specialization of ESCs.

#### Safeguarding genomic stability by the cohesin complex in ESCs

The rapid proliferation of ESCs necessitates sophisticated mechanisms to maintain genomic stability, in which the cohesin complex plays a dual regulatory function. On the one hand, the cohesin complex ensures mitotic fidelity by physically linking sister chromatids. On the other hand, the cohesin complex responds to replication stress by regulating DNA repair pathways. The knockdown of meiosis-specific subunits, namely, REC8 and STAG3, has been observed to result in replication fork arrest and an increase in the formation of RAD51 and replication protein A (RPA) foci, which implies the increased DNA replication stress [27]. Further in-depth study has demonstrated that cohesin deficiency leads to RPA-focus accumulation with defective HRR [10]. Notably, cohesin deficiency leads to DNA damage response that interferes with the expression and reprogramming of pluripotency genes, which reveals the multifaceted nature of the cohesin complex in genomic stability [11]. While RetSat ensures precise loading of the cohesin complex on chromosomes by interacting with SMC1A and the condensin subunit Nudcd2, thereby maintaining mitotic fidelity, its absence leads to a remarkable increase in the tumorigenicity of ESCs [86]. As such, the cohesin complex safeguards the genomic stability of ESCs through dual roles to serve as a physical scaffold and dynamically orchestrate DNA repair processes by bridging chromatin architecture with repair machinery.

#### The epigenetic regulation by the cohesin complex in ESCs

Chromatin modifications interacting with 3D structures are central to epigenetic regulation. A particularly prominent example of this interaction is the functional crossover of cohesin with polycomb repressive complex 1 (PRC1) in the fate determinations of ESCs. Wapal, a cohesin unloading factor, regulates loop dynamics by counteracting SMC3-mediated extrusion. Its depletion destabilizes PRC1-bound chromatin domains (e.g., Hox clusters), which leads to the derepression of pluripotency inhibitors like Otx2 [9]. This finding is further supported by the observation that the transcription factor E2f6 has been shown to act via PRC2 to silence genes (e.g., *Stag3* and *Smc1β*) during the transformation of ESCs to epiblast stem cells, which ensures the initiation of the somatic cellular program [87]. It is important to note that the cohesin complex modulates gene expression by counteracting polycomb-dependent chromosome interactions in ESCs and it does not disrupt interactions between super-enhancers. This disruptive activity is independent of CTCF and insulation, and it appears to repress gene expression through the polycomb system [88]. Taken together, these studies suggest that the cohesin complex plays a key role in shaping the epigenetic plasticity of ESCs by dynamically balancing activating and repressive epigenetic modifications.

Furthermore, the regulation of DNA methylation and demethylation processes serves to expand the epigenetic function of the cohesin complex. In the context of ESCs with cohesin subunit deletions (e.g., RAD21), the global hypermethylation of DNA has been observed to be associated with the elevated expression of DNMT3b, a hallmark of an epigenetic regulation that has the potential to promote differentiation by silencing pluripotency genes [10]. Conversely, Oplr16 has been shown to induce active demethylation by recruiting TET2 to the Oct4 promoter, thereby demonstrating that the cohesin complex can affect gene expression by an indirect regulation of dioxygenase activity [84]. This bidirectionally regulatory network suggests that the cohesin complex is not merely a passive substrate for epigenetic modifications but rather a dynamic regulator that is actively involved in the remodeling of the epigenetic landscape of ESCs.

#### Regenerative medicine of pluripotent stem cells by cohesin complex regulation

In the field of regenerative medicine, the dynamic regulation of the cohesin complex is closely related to the efficiency of stem cell differentiation. For instance, SMC3 expression is considerably elevated in pancreatic precursor-cell-derived induced pluripotent stem cells (iPSCs) in comparison to that in fibroblast-derived iPSCs. This suggests that SMC3, as a subunit of the cohesin complex, may indirectly regulate the process of cell differentiation of pluripotent stem cells including ESCs by affecting the chromatin structure and gene expression [89], which might have great applications in regeneration medicine for cell transplantation for human diseases.

#### The dynamic reprogramming of somatic cells to pluripotent stem cells by cohesin complex regulation

During somatic cell reprogramming into iPSCs, cohesin, a structural protein complex essential for chromatin organization, exhibits a context-dependent dual role. On the one hand, the cohesin complex facilitates the activation of pluripotency genes (e.g., *OCT4*) by maintaining the 3D chromatin architecture, e.g., E-P looping. On the other hand, excessive cohesin complex depletion triggers the DNA damage response, leading to cell cycle arrest and suppression of pluripotency networks, which reduces reprogramming efficiency [11]. This suggests a delicate balance in cohesin complex activity: the optimal level of cohesin supports chromatin remodeling for cell reprogramming to iPSCs, whereas severe loss of the cohesin complex compromises genomic integrity.

### The regulation of the cohesin complex in NSCs

The cohesin complex is involved in regulating the balance between the proliferation and differentiation of NSCs by dynamically maintaining the chromatin structure and genomic stability. PHF2 promotes the formation of TADs by binding to RAD21, maintains efficient replication initiation sites, and inhibits the activation of dormant replication to ensure normal DNA replication [14]. The imbalance of proliferation and differentiation of NSCs in 3D genomic regulation affects developmental programming. In early forebrain formation, the knockdown of STAG2 and SMC1A results in the obvious up-regulation of the expression of forebrain-development-related genes (e.g., *ZIC2* and *GLI2*), which may account for the etiology of holoprosencephaly [12].

At the cell cycle level, the overexpression of Gli1 causes significant induction of Gadd45a and down-regulation of *Cyclin A2* and *Stag1* messenger RNA, which involves G2–M arrest and cell apoptosis [90], suggesting that the dynamic expression of cohesin complex subunits plays a precise regulatory role in NSC proliferation and apoptosis. Concurrently, HNRNPA3 regulates chromosome segregation and mitotic progression in neural progenitors by interacting with SMC1A, and its deletion leads to delayed mitosis in these cells and affects the development of the cortex [13]. Furthermore, the distinct functions of STAG1 and STAG2 serve to refine the regulatory network. STAG1 is mainly involved in remote chromatin interaction of neurodevelopmental genes, whereas STAG2 regulates local loop structures near gene promoters [15]. Imbalance in the cohesin regulatory network may be implicated in the pathological processes of neuropsychiatric disorders by affecting the expression of key genes in NSCs, e.g., *TYW5* [91].

### The regulation of the cohesin complex in SSCs

The cohesin complex has been demonstrated to regulate the self-renewal, differentiation, and progression of SSCs through dynamic subunit assembly and chromatin interaction. Significantly, we have recently demonstrated that RAD21, a cohesin core subunit, forms a functional complex with the transcriptional coactivator YAP1 to regulate the transcriptional activity of NEDD4, thereby maintaining the self-renewal of human SSCs and inhibiting their apoptosis [92]. The loss of function of *Yap1*-conditioned knockout in this pathway leads to mitotic arrest and impaired spermatogenesis in mice [92], and abnormalities in the YAP1/RAD21/NEDD4 axis are associated with human non-obstructive azoospermia [92], highlighting new targets for clinical applications of the cohesin complex in treating male infertility. It is noteworthy that single-cell transcriptomic data have revealed that cohesin-related factors (e.g., *SMC3*) are specifically enriched in SSCs and they may regulate the transcription of germline-critical genes, e.g., *ZBTB16* (*Plzf*) and *DAZL*, by remodeling chromatin topology [93].

### The regulation of the cohesin complex in other stem cells

In intestinal stem cells (ISCs), the cohesin complex regulates the expression of the transcription factor Esg by maintaining its binding to the promoters of differentiation genes, thereby repressing the expression of differentiation-associated genes to retain stemness characteristics. The deletion of *RAD21* leads to aberrant activation of Esg target genes (differentiation-related genes), which induces ISCs to differentiate prematurely into intestinal epithelial cells. This process is independent of the Notch signaling pathway [94]. It is noteworthy that RAD21 displays a dual role in controlling ISC fate decisions. On the one hand, RAD21 enhances Lgr5 expression to maintain ISC self-renewal through a positive feedback loop of Wnt/β-catenin signaling. On the other hand, RAD21 mediates the loss of heterozygosity of *APC* genes, which exacerbates genomic instability [95].

In the context of epithelial–mesenchymal transition (EMT), RAD21 has been shown to impede the progression of EMT by repressing the transcription of *TGFB1* and *ITGA5* genes through the maintenance of their chromatin loop structure [96]. The down-regulation of RAD21 expression has been observed to result in the loosening of chromatin interaction, the promotion of mesenchymal phenotypes, and the acquisition of cancer stem cell properties. This 3D genomic regulation pattern is also critical in bone marrow stem cells. RAD21 haploinsufficiency impairs HRR, which leads to the accumulation of DNA damage after radiation and significantly reduces the regenerative capacity of gastrointestinal stem cells and bone marrow stem cells [97].

### Stem cell purity in the functions and regulation by the cohesin complex

It is important to note that different stem cell populations are inherently characterized by heterogeneity in terms of both phenotype and functions. This heterogeneity can be illustrated by fluctuations in pluripotency status and differences in the subpopulation differentiation tendencies of stem cells. The choice of purification methods has been shown to have a direct impact on the homogeneity of stem cells. The cohesin complex plays pivotal roles in regulating the topology of the genome by dynamically binding chromatin, while the functionality of this complex has been found to be highly dependent on the identities of stem cells. A large number of cell subpopulations makes it difficult to distinguish the functional priorities and molecular mechanisms of the cohesin complex. Consequently, the optimization and standardization of stem cell purification methodologies have the potential to reduce the impact of technical bias on the functional and mechanistic studies of the cohesin complex, thereby facilitating a more precise elucidation of its regulatory mechanisms in the dynamic reorganization of chromatin and the maintenance of genomic stability. Here, we further discuss the methodologies of cell purification for HSCs and ESCs.

For the purification of HSCs, the cell sorting strategy has been employed by using fluorescence-activated cell sorting by flow cytometry (FACS) or magnetic-bead-activated cell sorting using surface markers to enrich stem cells from heterogeneous cell populations. The 2 main subpopulations are Lin^−^CD34^+^CD38^−^ cells [61,72,98] and Lin^−^Sca1^+^c-Kit^+^ cells combined with signaling lymphocytic activation molecule (SLAM) molecules (e.g., CD150/CD48) [6,62,78]. Postsorting validation is similarly multifaceted, which includes FACS to examine the purity of HSCs (e.g., >95% CD34^+^ cells) and the colony-forming ability, tumorigenicity, and self-renewal capacity of stem cells by transplantation into immunodeficient mice [98]. For retaining the purity of ESCs, a basal medium is used to maintain its pluripotency [23,83] and to enrich the differentiated cell population (>95% Nestin/Sox2^+^) [80]. The general absence of an active purification approach may result in ineffective control of ESC heterogeneity; e.g., differentiation-prone cell residues may interfere with the functions of the cohesin complex in the dynamic remodeling of chromatin loops or the maintenance of pluripotency. Although no contradiction has been observed in cohesin complex functions due to differences in stem cell purification, distinct purification strategies and validation standards may be potentially interfering factors. Therefore, it is essential to employ appropriate cell sorting approaches for stem cell purification to improve our understanding of the regulation of the cohesin complex in stem cells.

### The interaction of the cohesin complex with other proteins in stem cell fate regulation

**Fig. 5.**
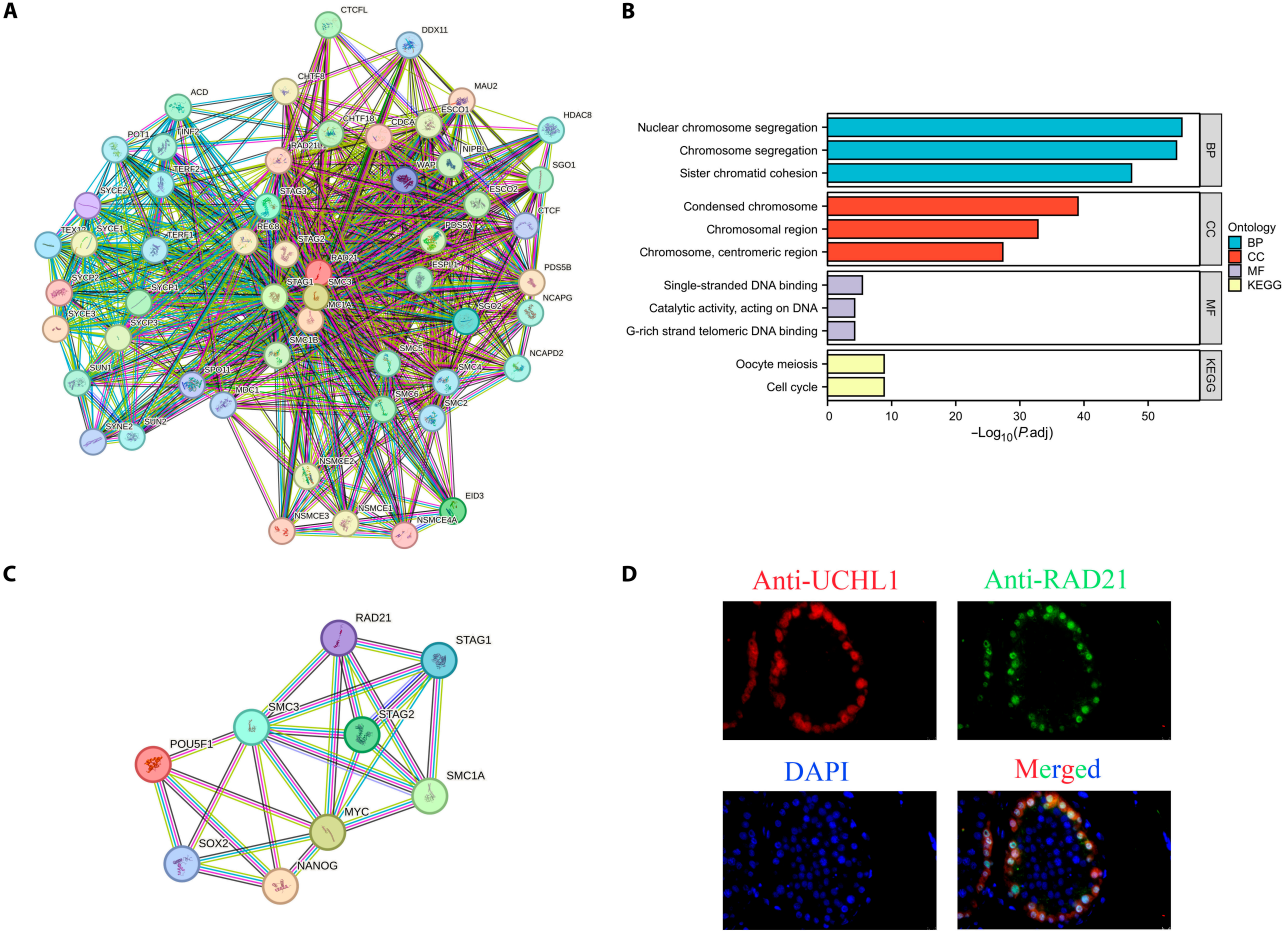
Gene networks, functional enrichment, and protein interaction of the cohesin complex in stem cells. (A and B) The gene interaction network of the 4 subunits of cohesin complex (SMC1, SMC3, RAD21, and STAG), which contains the genes *CTCF*, *CSM5*, and *SMC6* involved in sister chromatid cohesion, segregation, and DNA repair and the key nodes CTCF and SMC3 to maintain the stability of the network. The Gene Ontology (GO) and Kyoto Encyclopedia of Genes and Genomes (KEGG) enrichment analyses showed that cohesin-related genes are significantly enriched in the cell cycle, chromosome segregation (e.g., sister chromatid cohesion), and other processes and pathways, highlighting their important roles in the maintenance of the cell cycle and chromosome structure. (C) Interaction network of the subunits of the cohesin complex and pluripotency factors (e.g., POU5F1, NANOG, and SOX2) and stem cell proliferation (MYC). (D) RAD21 is colocalized with UCHL1 in human testicular tissues. BP, Biological Process; CC, Cellular Component; MF, Molecular Function; DAPI, 4′,6-diamidino-2-phenylindole.

We provide gene regulatory network analysis of cohesin complex in stem cell proliferation and differentiation (Fig. 5). As a crucial component in maintaining the chromosome structure, the cohesin complex is indispensable in ensuring chromosome stability, promoting sister chromatid segregation and coordinating the cell cycle (Fig. 5B). These fundamental functions are vital for preserving the genomic integrity of stem cells and safeguarding their capacity to divide and self-renew properly. The cohesin complex also interacts with stem cell pluripotency factors (e.g., POU5F1, SOX2, and NANOG) and a proliferation-related gene (*MYC*) (Fig. 5A and C). This interaction may influence the self-renewal and differentiation potential of stem cells by regulating the expression of specific genes, thereby directly participating in the process of determining their fate. Furthermore, our double immunostaining revealed the colocalization of RAD21, a cohesin subunit, with UCHL1, a hallmark of SSCs, in the human testes (Fig. 5D), which illustrates its role in stem-cell-specific fate determination. Overall, the cohesin complex plays a fundamental role in maintaining the biological functions of stem cells and directly participates in their fate regulation through a multilevel gene regulation and protein interaction network.

## The Association between Cohesin Complex Dysfunction and the Risk of Human Disorders

Cohesin gene mutations are associated with human diseases. The most notable disorders are Cornelia de Lange syndrome and Roberts syndrome. Cornelia de Lange syndrome, caused by mutations in genes like *NIPBL*, *SMC1A*, and *SMC3*, is characterized by phenotypic variation, e.g., craniofacial malformations and developmental delay. Roberts syndrome, resulting from the mutations of the *ESCO2* gene, which acetylates SMC3, manifests as growth retardation and skeletal developmental defects. Genes encoding cohesin subunits are mutated in a wide range of human cancers, with *STAG2* being the most commonly mutated subunit. These mutations are prevalent in bladder cancer, Ewing sarcoma, and myeloid malignancies. They can induce genomic instability, transcriptional dysregulation, aberrant cell proliferation, and differentiation, thereby promoting tumor development. Moreover, cohesin complex mutations are also found in other types of cancers, including glioblastoma, endometrial carcinoma, renal cell carcinoma, and breast cancer. Emerging therapeutic targets and agents are being investigated for cancers with cohesin complex mutations [5,99,100].

Diseases can be caused by or associated with cohesin complex mutations in stem cells. Mutations in the subunits of the cohesin complex in ESCs have been shown to be closely associated with the development of a variety of hematological malignancies. These mutations have been found to affect the differentiation homeostasis of ESCs by interfering with the 3D structure of chromosomes and the epigenetic regulatory network. In MDS, the deletion of the *STAG2* gene results in the aberrant expression of differentiation-related genes, which contributes to malignant cloning and transformation to AML. Clinical studies have demonstrated that *STAG2* mutations are markedly enriched in patients with MDS and AML, which is associated with an elevated risk of disease progression [60–63]. Cohesin complex mutations have been demonstrated to induce alterations in the binding capacity of critical transcription factors (e.g., ERG/GATA2/RUNX1) through the process of remodeling chromatin accessibility. This process has been supposed to ultimately result in the development of leukemia [65]. *SMC3* mutations works with GATA1s variants and causes acute megakaryoblastic leukemia [70].

In the context of neurological disorders, the deletion of *STAG2* and *SMC1A* has been observed to result in the aberrant expression of genes that are critical for forebrain development. This phenomenon may be associated with the occurrence of holoprosencephaly [12]. Furthermore, an imbalanced expression of cohesin complex subunits has been demonstrated to interfere with NSC cycle regulation and apoptotic processes. Imbalance in the cohesin regulatory network may be associated with the development of neuropsychiatric disorders [90,91]. Our study on SSCs have revealed the critical role of the cohesin complex. Abnormalities in the molecular pathway synergistically regulated by RAD21 and YAP1 can directly lead to impaired spermatogenesis, which provides a new direction in the mechanistic resolution of non-obstructive azoospermia [92]. It is noteworthy that the deletion of RAD21 in ISCs disrupts the balance of stemness maintenance and differentiation [94], which may exhibit a procarcinogenic effect. Concurrently, the down-regulation of RAD21 expression, which inhibits the EMT process by modulating the 3D structure of chromatin, promotes the acquisition of cancer stem cell properties [96], which then enhances tumor aggressiveness. Further studies are required to provide a more comprehensive understanding of the molecular characteristics of distinct cohesin subunit mutations and the association of their dysfunctions and mutations with specific diseases. This will facilitate the development of effective therapeutic strategies for cohesin-complex-related diseases.

## Conclusions and Perspectives

As a key regulator of the 3D spatial organization of chromatin, the cohesin complex is involved in numerous aspects of stem cell fate determinations through dynamic chromatin binding and dissociation. This complex comprises core subunits, e.g., SMC1, SMC3, RAD21, and STAG, and it dynamically remodels TADs through ATPase-driven chromatin loop extrusion, thus affecting the spatial arrangement and functional status of the genome. Overall, the regulatory patterns of the cohesin complex in different types of stem cells share the following common characteristics: Firstly, the cohesin complex has been shown to regulate the activity of pluripotent gene clusters with respect to the accessibility of differentiation-associated enhancers in a variety of stem cell types. This regulatory mechanism is characterized by its spatiotemporal specificity. During stem cell self-renewal, the cohesin complex is responsible for maintaining the undifferentiated state by stabilizing the chromatin-interaction network of key transcription factors. At the initiation stage of stem cell differentiation, degradation of specific subunits (e.g., RAD21) promotes the expression of lineage-specific genes by releasing repressive chromatin loops. Secondly, the cohesin complex frequently functions in conjunction with other pivotal transcription factors, epigenetic regulatory factors, and diverse molecules. For instance, they interact with transcription factors (e.g., OCT4 and NANOG) or collaborate with epigenetic regulatory complexes (e.g., PRC2) to regulate gene expression and chromatin status. Thirdly, the cohesin complex regulates chromatin structure and interactions, which influences various aspects, including the balance between self-renewal and differentiation of stem cells, metabolic state, DNA repair, and maintenance of genomic stability. Finally, pathological studies have further demonstrated that genetic mutations or aberrant expression of the cohesin complex subunits can disrupt chromatin dynamic homeostasis and lead to stem cell hyperproliferation or premature stem cell death, which provides a molecular basis for better understanding of cohesin-associated developmental disorders and tumorigenesis.

Future studies should focus on advancing the in-depth analyses of the cohesin complex in the stem cell field across a range of levels. At the molecular level, the key roles of proteins’ phosphorylation and acetylation in the dynamic regulation of cohesin have been reported. However, it is necessary to establish associations between aberrant proteins’ modifications under pathological conditions (e.g., dysregulation of CDK or HDAC in cancer or stem cell improper development) and cohesin dysfunction. In terms of technological innovations, ultrahigh-resolution live cell imaging could be used to resolve the real-time behavior of the cohesin complex in stem cell division, and single-cell multi-omics techniques, including single-cell RNA transcriptomics and proteomics, could be integrated to reveal its regulatory heterogeneity. Additionally, cryo-electronic microscopy can be employed to observe the ultrastructures of the cohesin complex at the atomic levels to further explore its functions and molecular mechanisms in controlling stem cell fate determinations. In terms of clinical applications, it remains to be a core challenge on how to achieve tissue-specific intervention of cohesin complex functions in stem cells, and the effectiveness of targeted therapy using the cohesin complex needs to be balanced with the risk of genomic stability. Furthermore, the analyses of the synergistic networks between the cohesin complex and other epigenetic regulators (e.g., polycomb complex PRC1/PRC2 and CTCF) would be helpful to build systematic regulatory frameworks for stem cell fate determinations. The advancement of these research directions can not only deepen our understanding of functions and mechanisms of the cohesin complex in controlling stem cell fate decisions but also offer novel approaches for regenerative medicine and disease treatment.
